# SIRT1-mediated FoxOs pathways protect against apoptosis by promoting autophagy in osteoblast-like MC3T3-E1 cells exposed to sodium fluoride

**DOI:** 10.18632/oncotarget.11573

**Published:** 2016-08-24

**Authors:** Xiaolong Gu, Dandan Han, Wei Chen, Limei Zhang, Qianyun Lin, Jian Gao, Séamus Fanning, Bo Han

**Affiliations:** ^1^ College of Veterinary Medicine, China Agricultural University, Haidian District, Beijing 100193, P R China; ^2^ UCD-Centre for Food Safety, School of Public Health, Physiotherapy and Sports Science, University College Dublin, Belfield, Dublin 4, Ireland

**Keywords:** apoptosis, autophagy, MC3T3-E1 cells, SIRT1, sodium fluoride

## Abstract

Fluorine may result in damage to teeth, bones and other body tissues, and is a serious public health problem. SIRT1 deacetylates FOXOs, which brings about apoptosis and autophagy promotion or suppression. Fluorine may induce cell apoptosis, however, the role of autophagy in apoptosis induced by fluorine is still poorly understood, and the interaction between SIRT1 and FOXOs should be further illustrated. Therefore, this study investigated the mechanisms underlying the NaF- induced apoptosis and autophagy in osteoblast-like MC3T3-E1 cells *in vitro* through activating or inhibiting SIRT1. *Via* RT-PCR, western blot, flow cytometry assays, fluorescence and laser confocal microscopy, it was found that NaF induced both cell apoptosis and autophagy. Results also showed that NaF up-regulated SIRT1 expression in a dose-dependent manner. The autophagy of MC3T3-E1 was also up- regulated indirectly whilst apoptosis was significantly attenuated when incubated with the SIRT1 activator SRT1720. When SIRT1 inhibitor Ex-527 was used, the latter effects were reversed. Furthermore, SIRT1 increased deacetylation of FoxO1 and promoted the up-regulation of its target substrate Rab7, as well as increase of Bnip3 which was substrate of FoxO3, and we hypothesize that these pathways may cause an increase in autophagic flux and a reduction in apoptosis. In conclusion, SIRT1-induced autophagy enhancement protects against fluoride-induced apoptosis through autophagy induction in MC3T3-E1 cells, which may be associated with a SIRT1-FoxO1-Rab7 axis and a SIRT1-FoxO3-Binp3 axis. The role of SIRT1 in selecting between cell survival and death provides a potential therapeutic strategy in fluorosis.

## INTRODUCTION

Fluorine is indispensable for teeth and bones since it can prevent enamel and root caries, as well as stimulate the formation of bones. Low levels of fluorine intake (0.8∼1.2 mg/L) are beneficial being frequently included in toothpaste; however, high intake levels (> 1.5 mg/L) may induce pitting corrosion in enamel, a phenomenon called dental fluorosis. In addition, it can cause alterations in bone structure leading to conditions such as osteoporosis and osteosclerosis. Therefore, fluorine in the environment has a dual role. On the one hand, fluorine is critical for protecting teeth and bones, and on the other hand, excess fluorine can result in damage to both along with other body tissues. Fluorosis remains a public health challenge world-wide, and there are endemic fluorosis districts in most areas in China [1].

Research data shows that fluorine may induce cell apoptosis via an oxidative stress dependent pathway, leading to an increase in lipid peroxidation in the cell, resulting in mitochondrial dysfunction and activation of downstream pathways [2, 3]. Individuals express anti-apoptosic genes as well as pro-apoptosic genes that can be induced by fluorine. It has been reported that there is a down-regulation of Bcl-2 in HGF (human gingival fibroblasts) exposed to 20 mM NaF, accompanied by the activation of the mitochondria-mediated cell death pathway including: (1) Cyt C release from the mitochondria to the cytoplasm, (2) activation of the caspase cascade pathway, (3) cleavage of Poly-(ADP-ribose) polymerase (PARP) and (4) expression of voltage dependent ionic channel [2]. Lee et al. [2] reported a second apoptosic pathway that was induced by fluorine. Fas Ligand (Fas-L) was reported to be up-regulated.

Few reports have been published describe the relationship between autophagy and fluorine-induced apoptosis. Autophagy is a catabolically driven process whereby stressed cells form cytoplasmic, double-layered, crescent-shaped membranes known as phagophores, mature into complete autophagosomes [4]. The autophagosomes engulf long-lived proteins and damaged cytoplasmic organelles to provide cellular energy and building blocks for biosynthesis [4]. The connection between apoptosis and autophagy is complex, and remains to be fully elucidated [5]. In some situations, autophagy is regarded as a rescue strategy to prevent apoptosis [6]. In contrast, autophagy may interact with apoptosis, or an alternative mechanism when apoptosic deficiencies arise, ultimately leading to cell death [5].

SIRT1 belongs to member of sirtuin family [7], it is a nicotinamide adenosine dinucleotide (NAD+) dependent deacetylase enzyme that functions to remove acetyl groups from histone and non-histone proteins [8]. SIRT1 has broad biological functions including gene silencing, stress resistance, apoptosis, inflammation and ageing [9]. *In vitro* experiments previously showed that SIRT1 could inhibit the activity of Bax, Ku70, FOXO and Rb (retinoblastoma) [10, 11]. In addition, it has been shown that SIRT1 may promote autophagy, possibly acting through related genes including Atg5, Atg7 and Atg8, and it regulates autophagy by means of deacetylating them [12].

Suzuki et al. [13] reported that SIRT1 may be involved in autophagy of LS8 cells previously induced by fluorine. In this case, autophagy was enhanced and apoptosis was alleviated after the cell was pretreated with RES (resveratrol). To date, reports describing the toxicological effects induced by fluorine are restricted to cell stress, cell cycle and apoptosis, and limited research describing the relationship between fluorine and autophagy exists. Thus, although SIRT1 is linked to autophagy as well as apoptosis, its definitive role it plays in the cell following fluoride exposure remains unclear. In the present study, we examined the inter connections between fluoride-induced autophagy and apoptosis in MC3T3-E1 cells, and identified a role of SIRT1 in selecting between cell survival and death, thereby providing new insight into the responses detected during fluorosis.

## RESULTS

### Assessment of apoptosis in osteoblast induced by NaF

**Figure 1:**
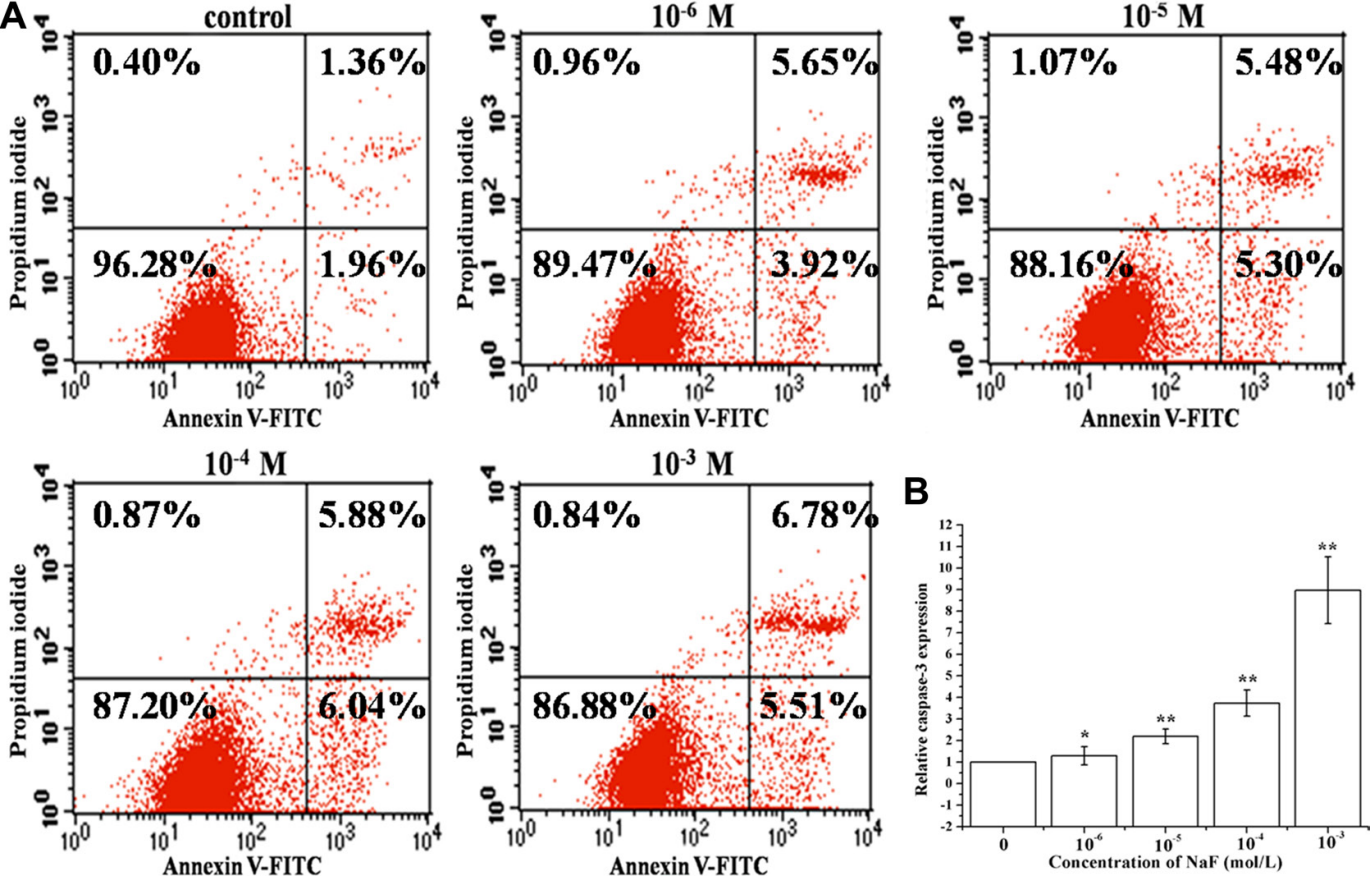
Assessment of apoptosis in cells treated with 10^–6^, 10^–5^, 10^–4^ and 10^–3^ mol/L NaF (**A**) The apoptotic rates were detected by FCM of annxin V-FITC/PI dual staining. Q1 quadrant (annexin V–, PI+) represented dead cells; Q2 quadrant (annexin V+, PI+) represented late apoptotic cells; Q3 quadrant (annexin V+, PI–) represented early apoptotic cells; Q4 quadrant (annexin V–, PI–) represented live cells. (**B**) The caspase 3 mRNA levels were detected using RT-PCR assay. Columns, mean of three independent experiments; mean ± SD; ^*^P < 0.05,^* *^P < 0.01; ^#^*P* < 0.05; ^##^*P* < 0.01. The same as below.

RT-PCR, FACS and FCM analysis of annexin V-FITC/PI dual staining were performed to detect apoptosis in cells treated with 10^–6^, 10^–5^, 10^–4^ and 10^–3^ mol/L NaF for 24 h. Annexin V-FITC/PI dual staining demonstrated that NaF induces a significant increase in the apoptotic rate [(Q2+Q3)%] (Figure 1A). Results also showed that caspase-3 mRNA expression level increased in a dose-dependent manner (Figure 1B). These data suggest that NaF induces caspase 3-mediated apoptosis in MC3T3-E1 cells.

### Assessment of autophagy in osteoblast induced by NaF

**Figure 2:**
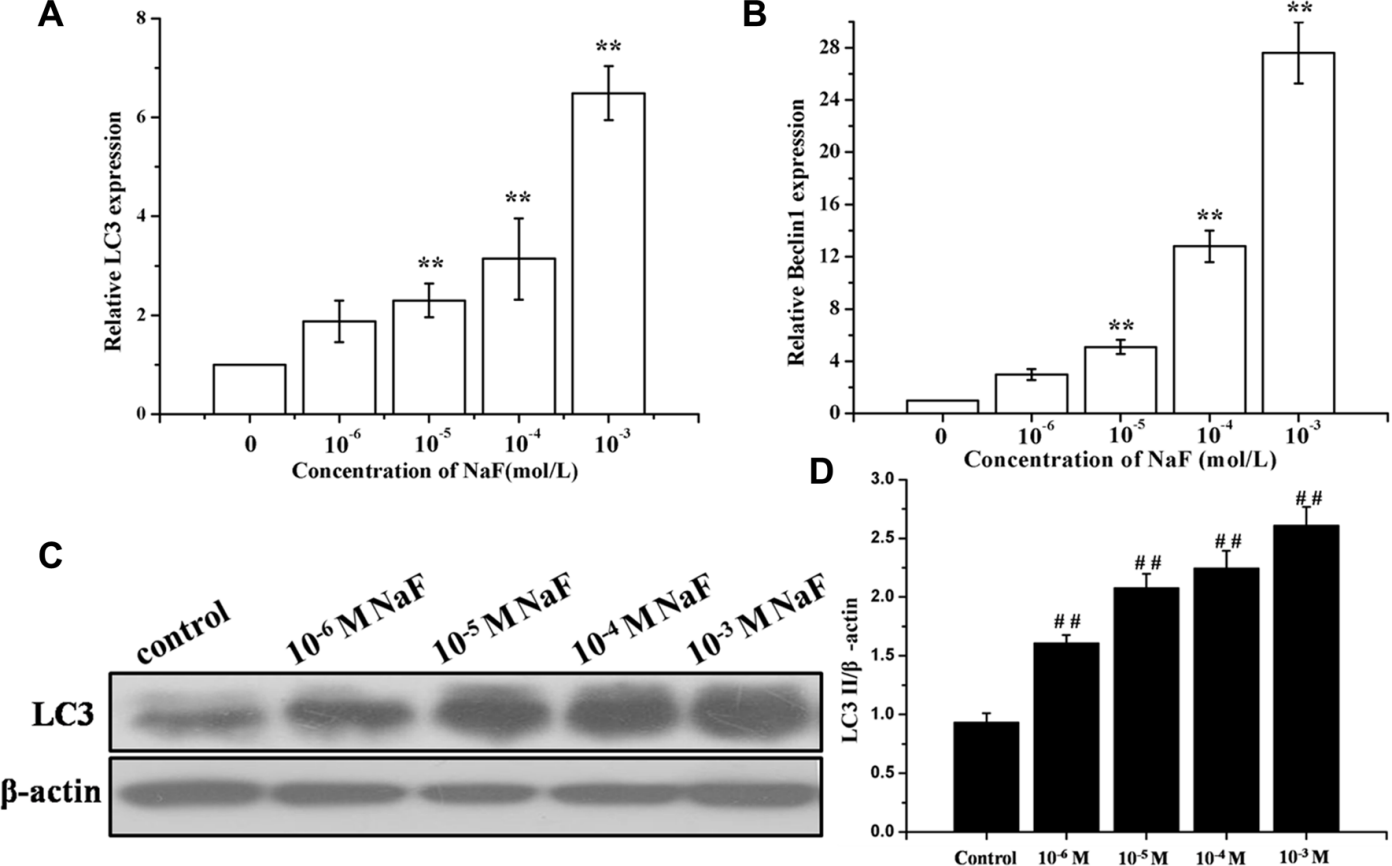
Assessment of autophagy in osteoblast induced by NaF (**A**) The LC3 mRNA levels were detected using RT-PCR assay. β-actin was used as an reference gene. (**B**) The Beclin1 mRNA levels were detected using RT-PCR assay. (**C**) The LC3-II, LC3-I protein levels of treated cells were detected using Western blot assay. (**D**) Results of densitometric analysis for Western blot.

RT-PCR and western blotting analysis of LC3 were performed to detect autophagy in cells treated with 10^– 6^, 10^–5^, 10^–4^ and 10^–3^ mol/L NaF for 24 h. Figure 2A and 2C showed that NaF significantly increased the expression of LC3 mRNA and proteins levels in osteoblasts. Meanwhile, the mRNA expression levels of Beclin 1 had accordant trend with LC3 (Figure 2B). These observations show that NaF induces autophagy of MC3T3-E1 cells in a dose-dependent manner.

### Detection of SIRT1 in osteoblast induced by NaF

**Figure 3:**
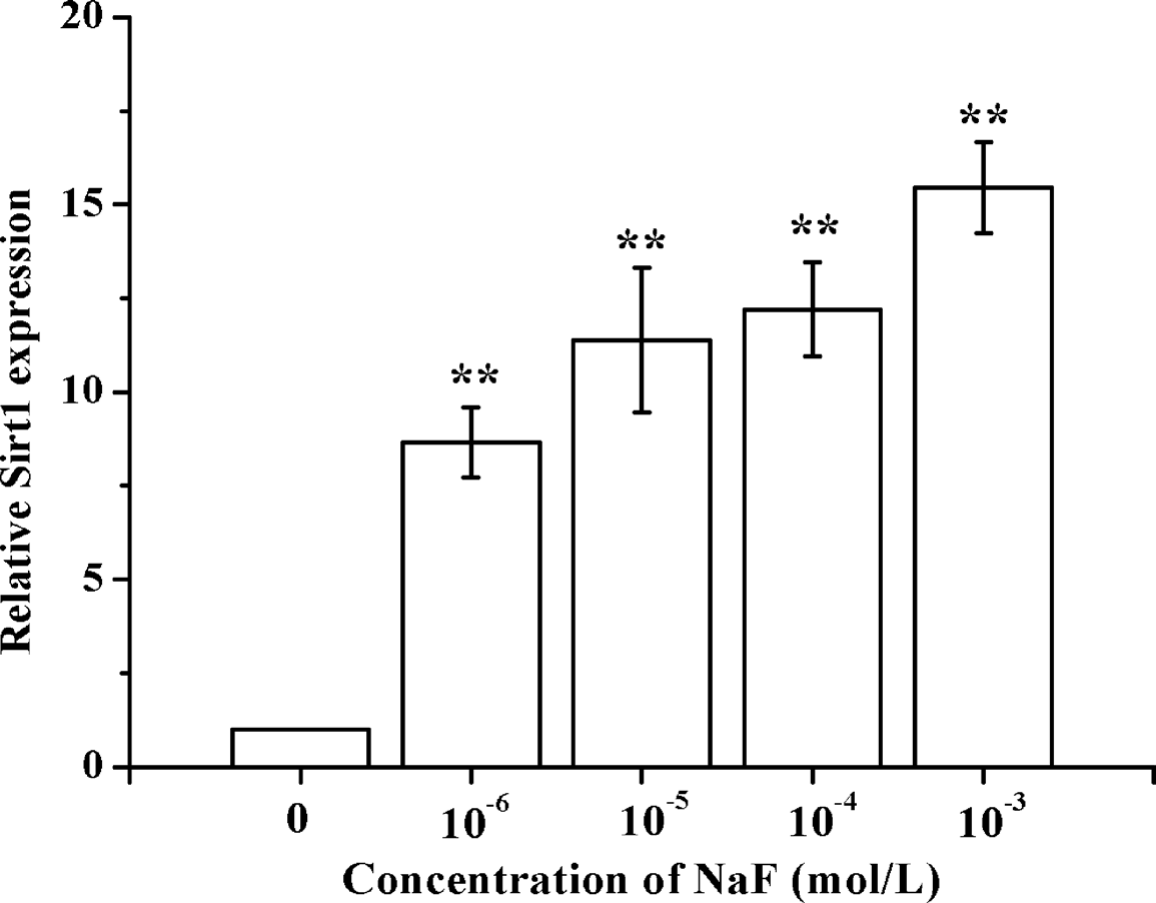
Determination of SIRT1 in osteoblast induced by NaF After the cells reached a steady-state of exponential growth in normal media, they were exposed to different concentrations of sodium fluoride for indicated time. And the SIRT1 mRNA levels were detected using RT-PCR assay.

Results showed that SIRT1 mRNA expression levels increased in a dose-dependent manner after treatment with various concentrations of NaF for 24 h (Figure 3).

### Effects of SIRT1 on cell viability of MC3T3-E1

**Figure 4:**
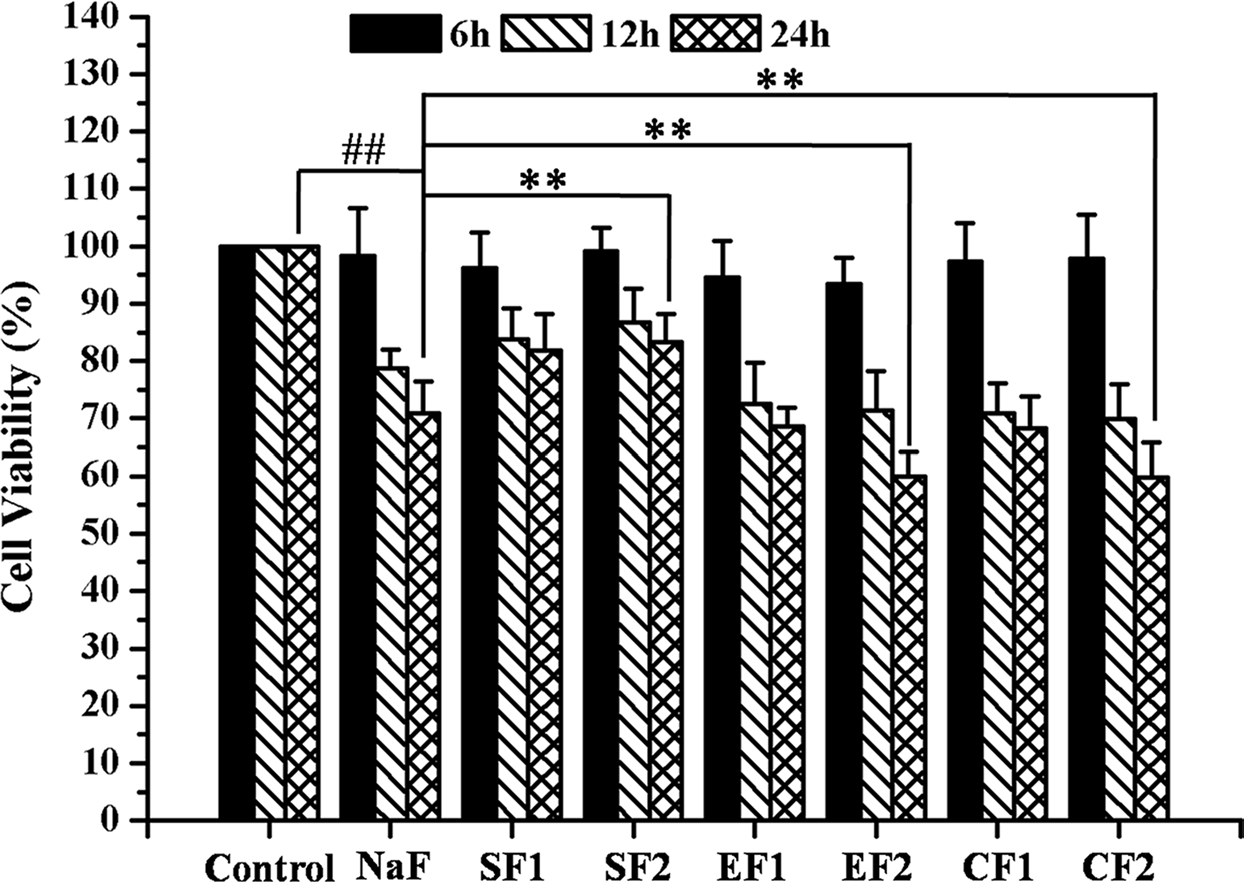
Effects of SIRT1 on cell viability of MC3T3-E1 exposed to NaF MC3T3-E1 cells were pretreated with SRT1720 or Ex-527 or CQ at indicated concentrations for 2 h, and then treated with 10^–4^ M NaF for 6 h, 12 h and 24 h. Viable cells were detected by MTT assay and cell viability (%) was calculated. The data were represented as mean ± SD from three independent experiments. Note: SF1: SRT1720 (100 nmol/L)+NaF, SF2: SRT1720 (200 nmol/L)+NaF, EF1: Ex-527 (400 nmol/L)+NaF,EF2: Ex-527 (800 nmol/L)+NaF, CF1: CQ (50 nmol/L)+NaF, CF2: CQ (50 nmol/L)+NaF.

**Figure 5:**
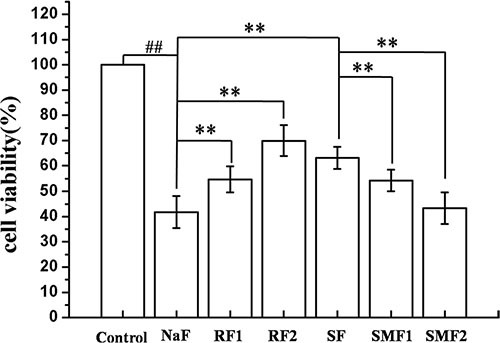
Effects of autophagy on cell viability of MC3T3-E1 exposed to NaF MC3T3-E1 cells were pretreated with Rapamycin or SRT1720 or SRT1720 combined with 3-MA at indicated concentrations for 2 h, and then treated with 10^–4^ M NaF for 48 h. Viable cells were detected by MTT assay and cell viability (%) was calculated. The data were represented as mean ± SD from three independent experiments. Note: RF1: Rapamycin (100 nmol/L)+NaF, RF2: Rapamycin (200 nmol/L)+NaF, SF: SRT1720 (200 nmol/L)+NaF, SMF1: SRT1720 (200 nmol/L)+3-MA (5 mmol/L)+NaF, SMF2: SRT1720 (200 nmol/L)+3-MA (10 mmol/L)+NaF.

MC3T3-E1 cells were pretreated with SRT1720 or Ex-527 or CQ at indicated concentrations for 2 h, and then treated with 10^–4^ M NaF for 6 h, 12 h or 24 h. As shown in Figure 4, NaF induced a notable inhibition of cell viability when compared with control. Pretreatment with 100 or 200 nM SRT1720 rescued the decreasing cell viability caused by NaF. In addition, the decrease of cell viability in osteoblasts pretreated with 400 or 800 nM Ex-527 or 50 or 100 μM CQ was aggravated compared with NaF-treated group. Furthermore, MC3T3-E1 cells were pretreated with Rapamycin or SRT1720 or SRT1720 combined with 3-MA at indicated concentrations for 2 h, and then treated with 10^–4^ M NaF for 48 h. As shown in Figure 5, pretreatment with 100 or 200 nM Rapamycin or 200 nM SRT1720 alleviated NaF-induced cell death, nevertheless, 3-MA abolished the alleviation induced by SRT1720. Therefore, the results above indicated that SIRT1 protected osteoblast from fluoride toxicity through autophagy induction.

### SIRT1 inhibited NaF-induced apoptosis

**Figure 6:**
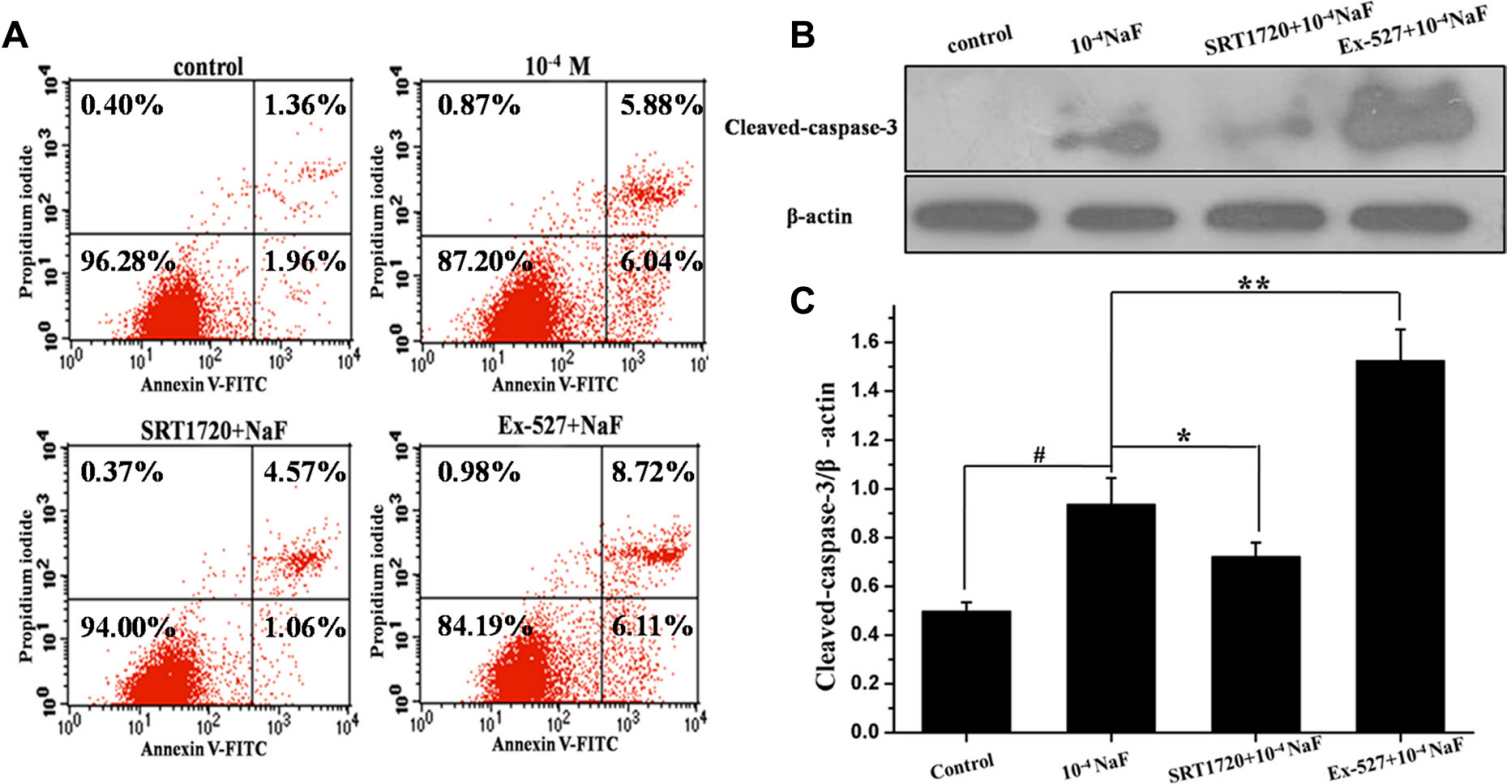
SIRT1 inhibited NaF-induced apoptosis FCM of annxin V-FITC/PI dual staining was performed and cleaved-caspase 3 protein expression level was detected to evaluate apoptosis in cells treated with NaF alone, pre-incubated with SRT1720 or pre-incubated with Ex-527. (**A**) The apoptotic rates were detected by FCM of annxin V-FITC/PI dual staining. (**B**) The cleaved-caspase 3 expression levels were detected using Western blot assay. (**C**) Results of densitometric analysis for Western blot.

FACS FCM analysis of annexin V-FITC/PI dual staining and western blot were performed to detect apoptosis in cells. When cells were treated with 10^–4^ mol/L NaF for 24 h, or with 10^–4^ mol/L NaF in the presence of 200 nmol/L SRT1720, or with 10^–4^ mol/L NaF in the presence of 800 nmol/L Ex-527 for 24 h. Annexin V-FITC/PI dual staining demonstrated that pretreatment with SRT1720 induces a significant decrease in the apoptotic rate [(Q2+Q3)%], and the apoptotic rates of cells increased after pretreatment with Ex-527 (Figure 6A), and cleaved-caspase-3 expression level decreased after pretreatment with SRT1720 when comparing with treatment of NaF alone. In contrast, caspase-3 expression levels increased significantly after pretreatment with Ex-527 (Figure 6B).

### SIRT1 enhanced NaF-induced autophagy

**Figure 7:**
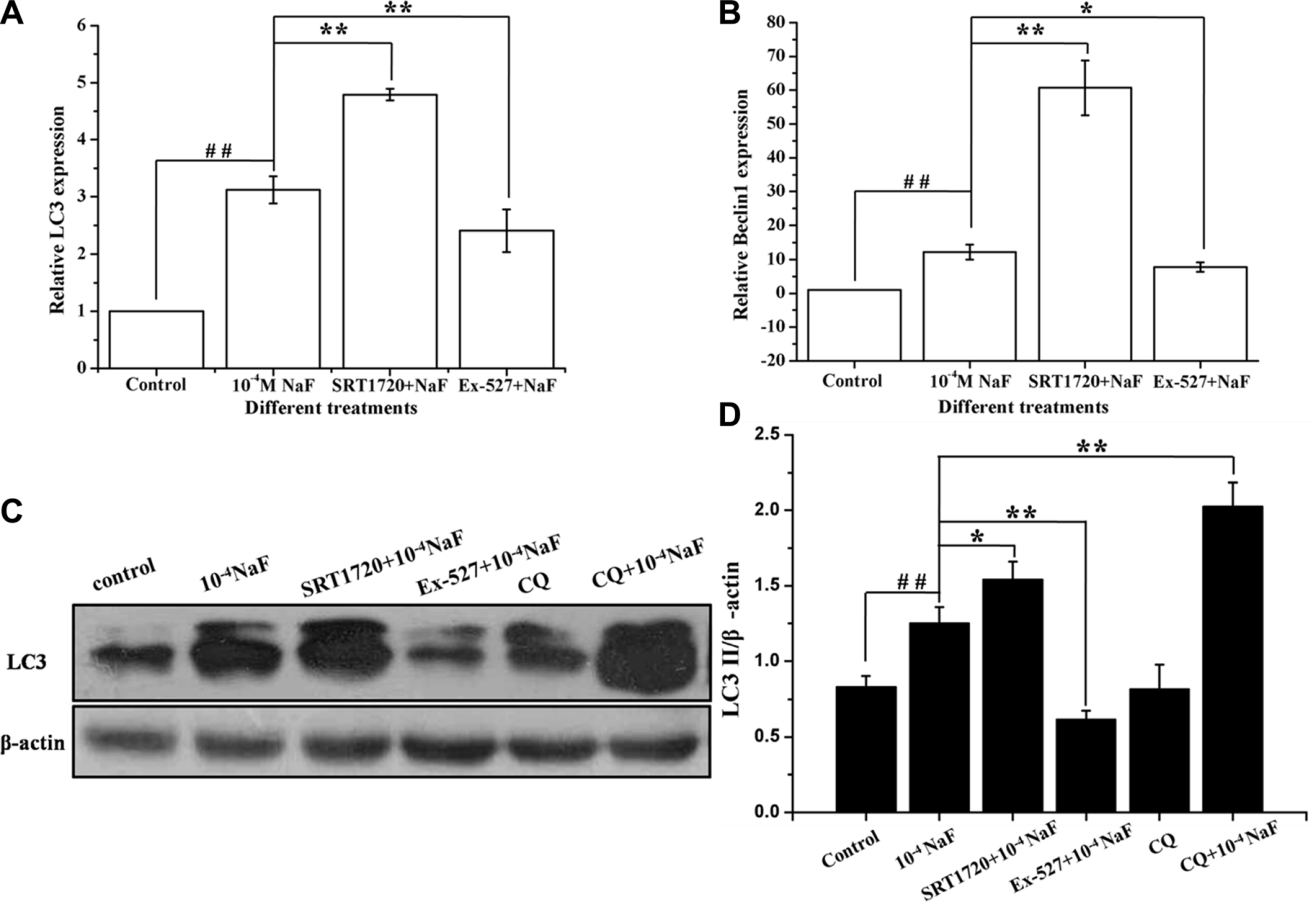
SIRT1 enhanced NaF-induced autophagy The LC3 protein and Beclin1 mRNA levels were detected in cells treated with NaF alone, pre-incubated with SRT1720 or pre-incubated with Ex-527 or pre-incubated with CQ. (**A**) and (**B**) After designed experiment measures, the LC3 and Beclin1 mRNA levels were detected using RT-PCR assay. (**C**) The LC3 protein levels of treated cells were detected using Western blot assay. (**D**) Results of densitometric analysis for Western blot.

RT-PCR, western blotting and fluorescence microscopic analysis of LC3 cells were performed to detect autophagy following treatment with NaF alone, or when pre-incubated with SRT1720 or pre-incubated with Ex-527. Data showed that LC3 mRNA and protein levels increased following pretreatment with SRT1720 when compared with treatment using NaF alone. In contrast LC3 mRNA and protein levels decreased after pretreatment with Ex-527, pretreatment of CQ increased LC3 expression level when compared with NaF treatment (Figure 7A and 7C). Meanwhile, the mRNA expression levels of Beclin 1 were consistent with LC3 (Figure 7B). The results above indicated that NaF induced autophagy flow, and autophagy was aggravated or alleviated with SRT1720 or Ex-527 pretreatment.

**Figure 8:**
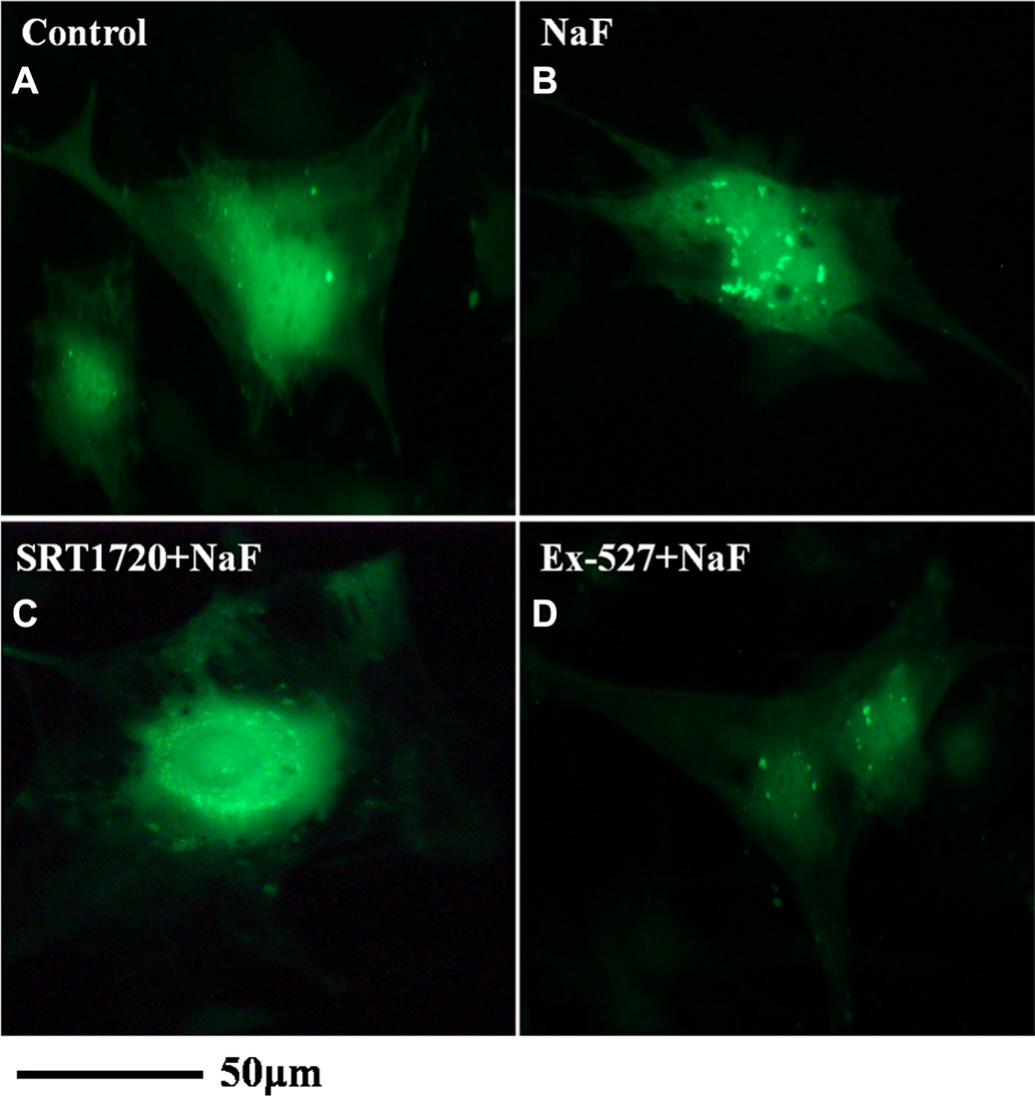
Fluorescence microscope analysis was performed to evaluate autophagy in cells treated with NaF alone, pre-incubated with SRT1720 or pre-incubated with Ex-527 (**A–D**) (200×). Localization of LC3-II at autophagosome membrane was indicated as green fluorescent punctate dots. The representative images are shown.

**Figure 9:**
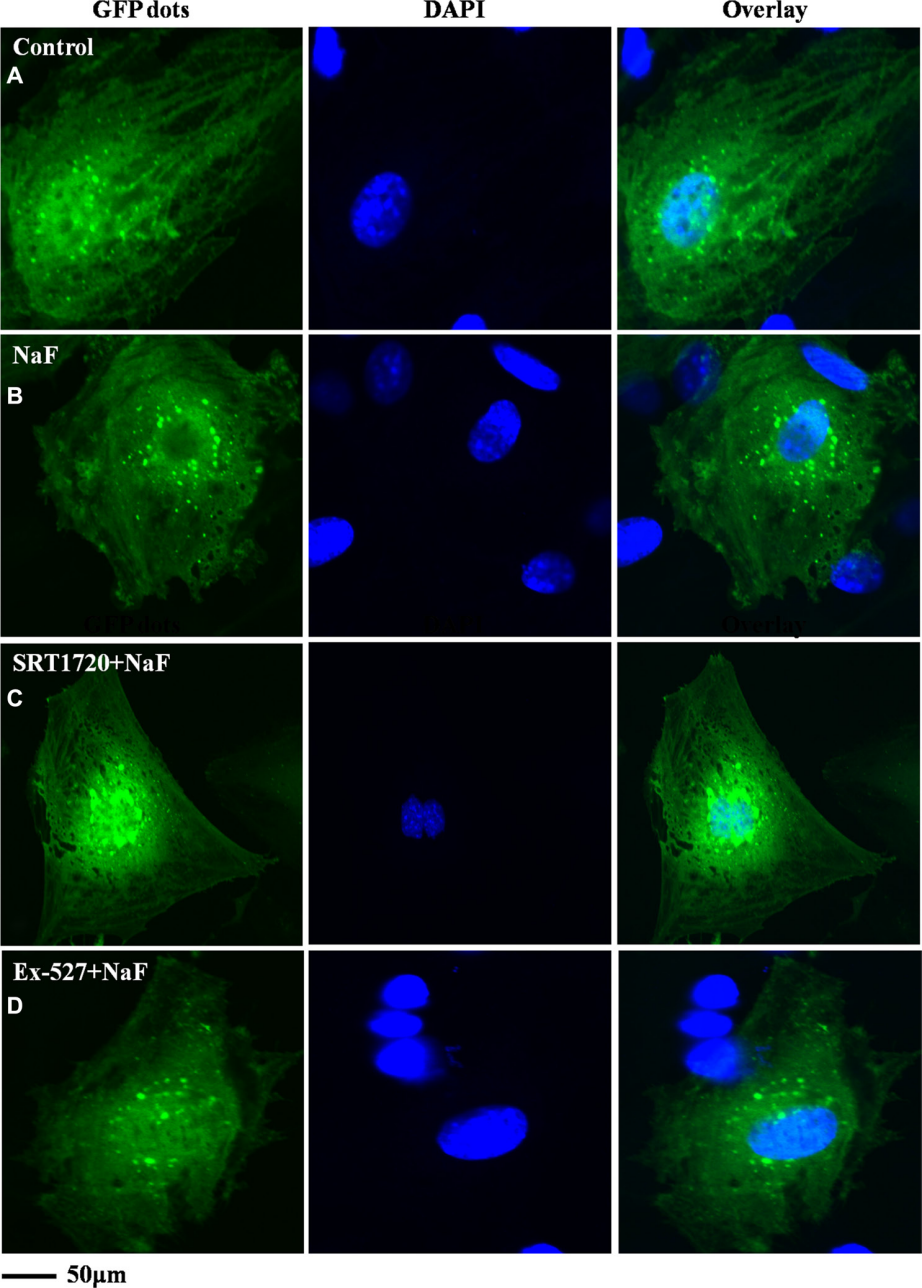
Confocal microscope analysis was performed to evaluate autophagy in cells treated with NaF alone, pre-incubated with SRT1720 or pre-incubated with Ex-527 (**A–D**) (400×). Representative images of fluorescent LC3 puncta after Ad-tf-LC3 transduction are shown.

Fluorescence and confocal microscopy data showed that numbers of green fluorescent punctate increased in osteoblasts after pretreatment with SRT1720 when compared with treatment of NaF alone (Figure 8B, 8C and Figure 9B, 9C). Numbers of punctate decreased in osteoblasts after pretreatment with Ex-527 (Figure 8D and Figure 9D).

### The pathways mediated by SIRT1 in NaF-induced apoptosis and autophagy

**Figure 10:**
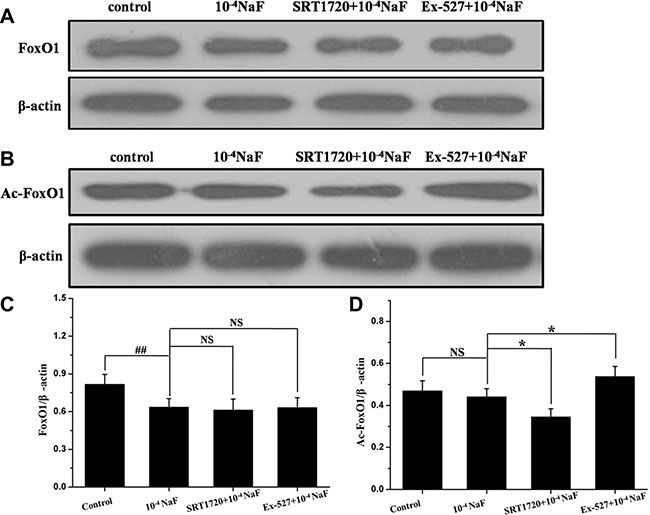
The pathways mediated by SIRT1 in NaF-induced apoptosis and autophagy The protein expression levels of FoxO1 and Ac-FoxO1 were detected to evaluate SIRT1-mediated pathways in cells treated with NaF alone, pre-incubated with SRT1720 or pre-incubated with Ex-527. (**A, B**) The FoxO1 and Ac-FoxO1 expression levels were detected using Western blot assay. (**C, D**) Results of densitometric analysis for Western blot.

**Figure 11:**
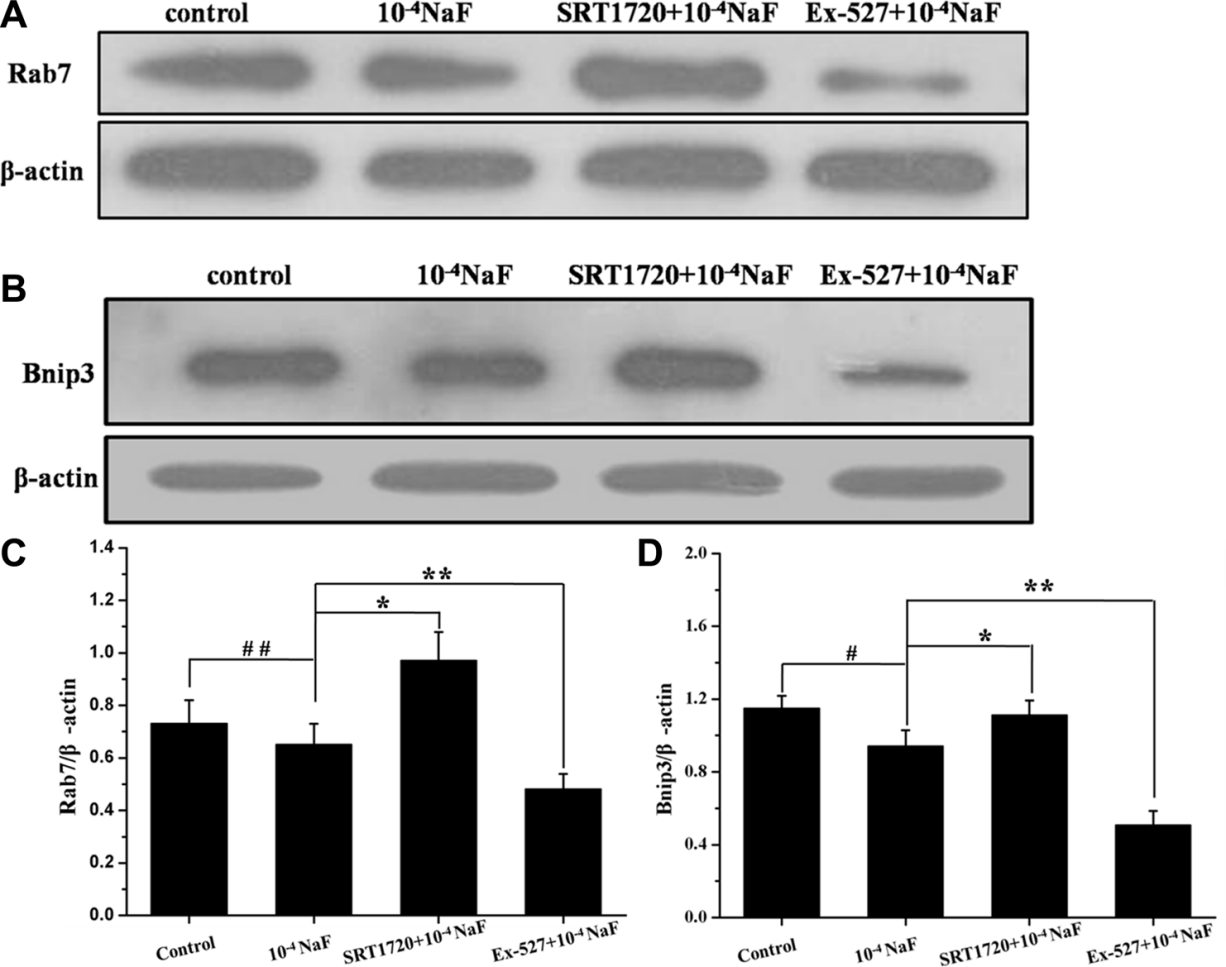
The pathways mediated by SIRT1 in NaF-induced apoptosis and autophagy The protein expression levels of Rab7 and Binp3 were detected to evaluate SIRT1-mediated pathways in cells treated with NaF alone, pre-incubated with SRT1720 or pre-incubated with Ex-527. (**A, B**) The Rab7 and Binp3 expression levels were detected using Western blot assay. (**C, D**) Results of densitometric analysis for Western blot.

The protein expression levels of FoxO1, Ac-FoxO1, Rab7, Bnip3 were detected by western blot in the presence or absence of Ex-527 or SRT1720 following treatment with NaF (Figures 10 and 11). The protein expression levels of FoxO1 and Ac-FoxO1 were suppressed after NaF treatment, and FoxO1 expression had no significance between different treatments, while Ac-FoxO1 increased or decreased in Ex-527 or SRT1720 pretreated group when compared with NaF treated group (Figure 10A and 10B). Data also showed that Rab7 and Bnip3 protein levels increased following pretreatment with SRT1720 when compared with treatment using NaF alone. In contrast Rab7 and Bnip3 protein levels decreased significantly after pretreatment with Ex-527 (Figure 11A and 11B). The results above indicated that the protection of SIRT1 against fluoride-mediated cell death may be associated with SIRT1-FoxO1-Rab7 axis, SIRT1-FoxO3-Binp3 axis.

## DISCUSSION

Accumulating evidence revealed that autophagy and apoptosis could either cooperate or antagonize each other, thus differentially influencing the fate of the cells. In this study we examined whether FOXOs participate in the effect of SIRT1 modulators in osteoblast-like MC3T3-E1 cells exposed to fluoride. Firstly, we showed that NaF treatment led to significant apoptosis in MC3T3-E1 cells *via* caspase-3 activation, a feature that could be verified by Annexin V-FITC/PI staining. In general, caspase-3 is a key and common protease in both mitochondria- and death receptor-dependent pathways [14, 15]. Previous studies reported that fluoride induces apoptosis in human leukemia HL-60 cells by activating caspase-3 [1, 16]. Interestingly, we also found the increase of Beclin1 mRNA and LC3 II protein levels, thus confirming the presence of autophagy in the osteoblasts exposed to NaF. Correspondingly, an increase of SIRT1 mRNA expression levels in a concentration-dependent pattern also been observed. These data suggested that the increase in autophagy was associated with an increase in SIRT1 protein levels, which is consistent with the observations reported by Suzuki [13]. The anti-apoptotic function of SIRT1 was self-explanatory [17–19]. However, the role of SIRT1 in autophagy is controversial. Some studies reported that SIRT1 promotes autophagy by down-regulating mTOR signaling [20]; whilst, others stated that the inhibition of SIRT1 activity augmented autophagy [21]. The present study demonstrated that SIRT1 protected osteoblast from fluoride toxicity through autophagy induction, which was verified by MTT using the known SIRT1 activator SRT1720 and the inhibitor Ex- 527, Pretreatment with Rapamycin or SRT1720 alleviated fluoride cytotoxicity, Ex-527 or CQ exerted the opposite effect, CQ verified that the autophagy inhibition could aggregate the NaF-induced decrease in cell viability, nevertheless, 3-MA abolished the alleviation induced by SRT1720. It was further investigated whether the up-regulation of autophagy mediated by SIRT1 could attenuate apoptosis of osteoblasts induced by fluoride. Our findings suggest that an increase in apoptosis rate induced by NaF could be alleviated by SRT1720, similarly, the apoptosis aggravated using an appropriate dose of Ex- 527, and the protein expression of cleaved-caspase-3 concomitantly decreased or increased in line with the corresponding treatment. Meanwhile, the expression of the autophagy marker protein LC3, fluorescence and confocal microscopy demonstrated the opposite effect. These findings suggested that autophagy activated by SIRT1 directly contributes to the survival of the MC3T3 cells. Suzuki [13] reported that the SIRT1 activator resveratrol (RES) increased autophagy, inhibited apoptosis, and decreased fluoride cytotoxicity in fluoride treated LS8 cells. Overall, SIRT1 protected against fluoride-mediated cell death.

**Figure 12:**
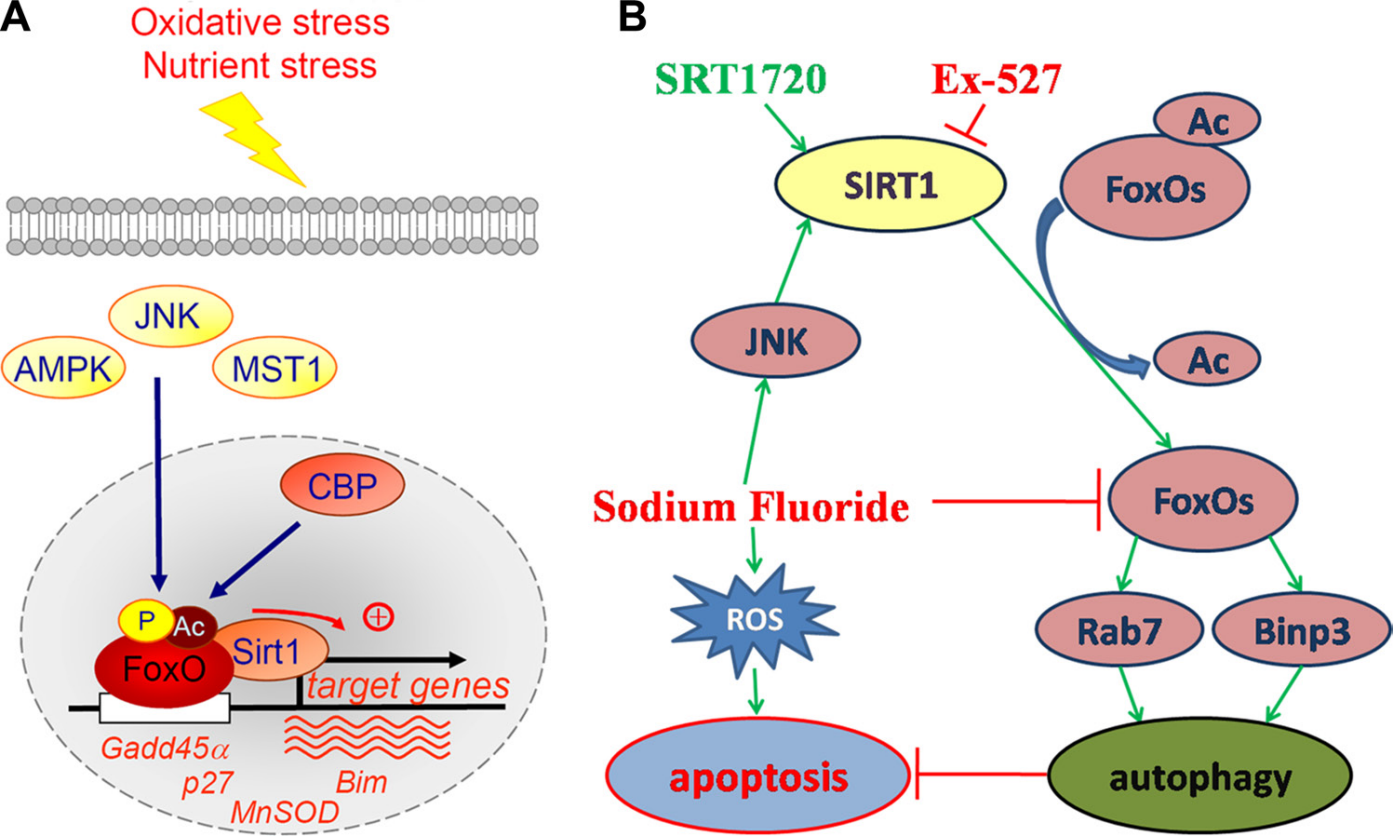
Positive regulation of FoxO factors by oxidative stress stimuli and the proposal signaling pathway of the current study (**A**) Oxidative stress induces the phosphorylation, acetylation and monoubiquitination of FoxO factors at a number of regulatory sites by several factors. In response to oxidative stress, FoxO factors translocate to the nucleus and bind to the deacetylase SIRT1, and specific genes involved in cell-cycle arrest and the response to stress are recruited. p27, cyclin-dependent kinase inhibitor; MnSOD, manganese superoxide dismutase; Bim, pro-apoptotic Bcl2-interacting mediator of cell death; Gadd45α, growth arrest- and DNA damage-inducible gene 45 α. (Dervis A M Salih and Anne Brunet./Curr Opin Cell Biol. 2008; 20(2): 126–136). (**B**) Fluoride induces ROS generation resulting in cell apoptosis. Meanwhile, fluoride activates SIRT1 through the JNK/c-Jun pathway. Activation of SIRT1 protects against fluoride-induced osteoblast apoptosis probably through two following mechanisms: SIRT1-FoxO1-Rab7 axis and SIRT1-FoxO3-Binp3 axis via autophagy promotion.

Suzuki et al reported that fluoride induces ROS generation that elicits JNK/c-Jun signaling, which result in oxidative damage, mitochondrial damage, DNA damage, and apoptosis. In contrast, fluoride activates SIRT1/autophagy as an adaptive response through the ROS-mediated JNK/c-Jun pathway to protect cells from fluoride-induced oxidative damage (Figure 12B) [22]. The results reported by Maiko Suzuki support our study. With respect to the potential mechanisms of SIRT1-regulated MC3T3-E1 cell survival, we hypothesized that SIRT1 may exert its effects via an increase in its interaction with the FOXO family members, which are implicated in regulating certain fundamental cellular functions. FoxO1 and FoxO3, belong to the FOXO family, are predominant FOXO isoforms in mammals, and they may promote autophagy and cardiomyocyte survival under oxidative stress conditions [23, 24]. Acetylation of FOXO family proteins results in a reduction in their transcriptional and biological activities, which could be reversed by SIRT1. The effects of SIRT1 on FOXO function varies depending on the target genes [25, 26]. However, the consensus which emerges is that SIRT1, acts by deacetylating FOXO factors, may play a crucial role in shifting the balance of cell death and stress resistance via preventing FOXO factors from inducing apoptosis [25, 27, 28]. FoxO1 and FoxO3 enhance autophagy in skeletal and cardiac muscles by activating genes that are involved in autophagosome [23, 29]. Our results showed that the protein expression levels of Ac-FoxO1 after treatments with SRT1720 or Ex-527 were decreased or increased, which indicated that SIRT1 suppressed FOXO1- induced cell apoptosis via its deacetylating function. Interestingly, FoxO1 activation was shown to increase the expression of Rab7, a small GTPase that facilitates late autophagosome-lysosome fusion. Furthermore, SIRT1 role in deacetylating FoxO3 leads to enhance expression of pro-autophagic Binp3. In this study, the trend in protein expression levels Rab7 and Binp3 was consistent with role proposed for SIRT1 after treatments with SRT1720 or Ex-527. Meanwhile, it should be noted that FoxOs pathways were suppressed by fluoride, although autophagy in osteoblast was upregulated, the efferocytosis of cell was not reverse.

In conclusion, we demonstrated that both apoptosis and autophagy increased in fluoride-induced MC3T3-E1 cells. These data also showed that autophagy was up-regulated by an appropriate dose of the SIRT1 activator SRT1720, and that the efferocytosis of apoptotic MC3T3-E1 was significantly attenuated when compared with SIRT1 inhibitor Ex-527. Furthermore, SIRT1-mediated deacetylation of FoxOs controls the SIRT1-FoxO1-Rab7 axis and SIRT1-FoxO3-Binp3 axis to alleviate the cytotoxicity induced by fluoride. The regulation of FoxO factors by stress and the proposal signaling pathway of the current study were shown in Figure 12A and 12B. These findings suggest an important role for SIRT1 in modulating cellular mechanisms and therefore may have direct implications in fluorosis diseases and possibly offer potential therapeutic strategies.

## MATERIALS AND METHODS

### Reagents and antibodies

Sodium fluoride, thiazolyl blue tetrazolium bromide (MTT), chloroquine (CQ) (autophagy inhibitor), dimethyl sulfoxide (DMSO), rapamycin (autophagy activator) and 3-MA (autophagy inhibitor) were purchased from Sigma-Aldrich. The SIRT1 activator SRT1720 and inhibitor Ex- 527 were purchased from Selleck Chemicals. Rapamycin and CQ were dissolved in PBS, 3-MA, SRT1720 and Ex-527 were dissolved in DMSO to provide a working stock solution. The DMSO concentration was maintained at 0.1% in all cell cultures, and it did not exert any detectable effect on cell growth nor cell death. Anti-LC3, anti-cleaved-caspase-3 and anti-FoxO1 antibodies were purchased commercially (Cell signal technology, CA, USA). Anti-Ac-FoxO1, HRP-conjugated goat anti-mouse and goat anti-rabbit antibodies were purchased from Santa Cruz Biotech Company. Anti-Bnip3 and anti-Rab7 were purchased from Abclonel Company.

### Cell culture and treatments

Murine osteoblast MC3T3-E1 cells were purchased from American Type Culture Collection (ATCC). These cells were maintained in α-MEM medium (Gibco) supplemented with 10% fetal bovine serum (FBS) (Hyclone) with 1% penicillin-streptomycin under 5% CO_2_ humidified atmosphere at 37 °C. NaF (Sigma, CA, USA) was dissolved in distilled α-MEM and sterilized through a 0.2 μm filter. After the cells reached a steady-state of exponential growth in normal media, they were exposed to different concentrations of sodium fluoride for indicated time.

### MTT assay

A 100-μL suspension of MC3T3-E1 cells was incubated in 96-well plates with or without the pretreatment of SRT1720 at defined concentrations (200 and 400 nmol/L) or Ex-527 at various concentrations (400, 800 nmol/L) or CQ at various concentrations (50, 100 μmol/L) for 2 h, and then treated with 10^–4^ M NaF for 6 h, 12 h or 24 h. In addition, MC3T3-E1 cells were pretreated with rapamycin (100 and 200 nmol/L) or SRT1720 or SRT1720 combined with 3-MA (5 and 10 mmol/L) at indicated concentrations for 2 h, and then treated with 10^–4^ M NaF for 48 h. After incubation for the defined period of time, MTT was added to each well at a final concentration of 0.5 mg/mL for 4 h, and the resulting formazan crystals were dissolved in formazan dissolving solution. The absorbance of these samples was recorded using a microplate reader (Bio-rad, California, USA) at 490 nm.

The growth inhibitory ratio was calculated as follows:

Growth inhibitory ratio (%) = [(A_control_– A_blank_ of control) – (A_sample_– A_blank_ of sample)] / (A_control_– A_blank_ of control) × 100%.

### RNA purification, cDNA synthesis and real time PCR

Total RNA was purified by homogenizing cells in Trizol (TransGen Biotech, Inc. Beijing, China) according to the manufacturer's instructions. The cDNA strand was synthesized from the purified RNA using the iScript cDNA synthesis kit (TransGen, Beijing, China), following the manufacturer's instructions. Real-time PCR was performed on a Roche Light Cycle system (Roche) with SYBR green PCR master mix (TransGen, Beijing, China) and 1 μL of first-strand cDNA as template with specific primers for SIRT1, caspase-3, LC3, Beclin1 which were shown in Table 1. The levels of gene expression were determined relative to that of β-actin.

**Table 1:** Prime and probe sequences with corresponding PCR product size and accession

Gene	Genbank accession no.	Primers and Probes (5′ to 3′)	Product (bp)
SIRT1	NM_019812.2	Forward: AATATATCCCGGACAGTTCCAGCC Reverse:ATCCTTTGGATTCCTGCAACCTGC	132
Caspase-3	NM_001284409.1	Forward: GATGTGGACGCAGCCAACCTCA Reverse: TCCGGCAGTAGTCGCCTCTGAA	247
LC3	NM_026160.4	Forward: CTTCTTCCTCCTGGTGAATGG Reverse: ATTGCTGTCCCGAATGTCTC	135
Beclin1	NM_019584.3	Forward: CCAATGTCTTCAATGCCACCTTC Reverse: GGCAGCATTGATTTCATTCCAC	119
β-actin	NM_007393.4	Forward: ATGCTCTCCCTCACGCCATCCT Reverse: ATCGGAACCGCTCGTTGCCAAT	264

### Analysis of apoptosis using the FCM of AV/PI dual staining

For annexin V-FITC/PI dual staining, cells were processed with an Annexin V-FITC kit purchased from Beyotime Institute of Biotechnology (Jiangsu, China) and deployed according to the manufacturer's instructions. The analysis of apoptotic cells was performed using flow cytometry (BD FACS Calibur, New Jersey, USA) at a low flow rate and a minimum of 1 × 10^4^ cells. Different subpopulations were distinguished using the following criteria: Q1, annexin V-negative, but PI-positive (ie, necrotic cells); Q2, annexin V/PI-double positive (ie, late apoptotic cells); Q3, annexin V/PI-double negative (ie, live cells); Q4, annexin V-positive, but PI-negative (ie, early apoptotic cells). The apoptotic rate was determined as the percentage of Q2+Q4.

### Protein extraction and western blot analysis

After MC3T3-E1 cells were lysed in a NP-40 lysis buffer (30 mM Tris-Cl, pH 7.5, 1 mM EDTA, 150 mM NaCl, 1% NP-40, 1 mM PMSF, and protease inhibitor mixture containing 1 μg/mL aprotinin and leupeptin), the extracts were centrifuged, and the cleared supernatants containing total protein were collected. After extraction, the protein concentration was determined using the BCA protein assay (Solarbio, Beijing, China) and an equal amount of protein was subjected to SDS-polyacrylamide gel electrophoresis prior to electro-transfer to PVDF membranes (Millipore, MA, USA). After blocking with 5% non-fat milk, the membranes were incubated with the designated primary and secondary antibodies, developed by the enhanced chemiluminescence method (Byotime, Jiangsu, China) and visualized using the Kodak Image Station. The band density was quantified using the Image J image processing program.

### Fluorescence and laser confocal microscopy

The method to evaluate tandem fluorescent LC3 puncta using Ad-tf-LC3 has been described previously [29]. Osteoblast cells were transfected with Ad-tf-LC3 at a ratio of 30 MOI. Twenty-four hours after adenovirus transduction, the cells were washed with PBS, treated with indicated drug, then fixed with 4% paraformaldehyde (PFA), mounted with a reagent containing 4′,6-diamidino-2-phenylindole (DAPI) (Byotime, Jiangsu, China), and viewed under a fluorescence microscope (Olympus IX70, CA, USA) and a laser confocal microscope (Olympus IX81, CA, USA). The number of GFP dots in treated groups was compared with that in control group.

### Statistical analysis

All data are presented as mean ± standard deviation (SD) for three independent repeats of each experiment. Statistical significance was determined using one-way Analysis of Variance (ANOVA) by SPSS 19.0 with *P*-values < 0.05 representing significance.
